# Tracing Nutrient Flux Following Monocarboxylate Transporter-1 Inhibition with AZD3965

**DOI:** 10.3390/cancers12061703

**Published:** 2020-06-26

**Authors:** Marta Braga, Maciej Kaliszczak, Laurence Carroll, Zachary T. Schug, Kathrin Heinzmann, Nicoleta Baxan, Adrian Benito, Gabriel N. Valbuena, Stephen Stribbling, Alice Beckley, Gillian Mackay, Francesco Mauri, John Latigo, Chris Barnes, Hector Keun, Eyal Gottlieb, Eric O. Aboagye

**Affiliations:** 1 Division of Cancer, Imperial College London, Hammersmith Hospital Campus, Du Cane Road, London W12 0NN, UK; m.braga13@imperial.ac.uk (M.B.); maciejkalis@yahoo.fr (M.K.); laurencecarroll@outlook.com (L.C.); kathrin_heinzmann@hotmail.com (K.H.); a.benito-mauricio@imperial.ac.uk (A.B.); gabriel@well.ox.ac.uk (G.N.V.); stephenstribbling@hotmail.com (S.S.); a.beckley11@gmail.com (A.B.); f.mauri@imperial.ac.uk (F.M.); j.latigo@imperial.ac.uk (J.L.); chris.barnes@imperial.ac.uk (C.B.); h.keun@imperial.ac.uk (H.K.); 2 Cancer Metabolism Research Unit, Cancer Research UK Beatson Institute, Switchback Rd, Glasgow G61 1BD, UK; zschug@wistar.org (Z.T.S.); g.mackay@beatson.gla.ac.uk (G.M.); eyalgot50@gmail.com (E.G.); 3 Molecular and Cellular Oncogenesis, Wistar Institute, 3601 Spruce Street, Philadelphia, PA 19104, USA; 4 Biological Imaging Centre, Imperial College London, Hammersmith Hospital Campus, Du Cane Road, London W12 0NN, UK; n.baxan@imperial.ac.uk

**Keywords:** monocarboxylate transporter 1, cancer metabolism, lactate, glycolysis, AZD3965, diffuse large B-cell lymphoma, metabolic flux, positron emission tomography (PET)

## Abstract

The monocarboxylate transporter 1 (MCT1) is a key element in tumor cell metabolism and inhibition of MCT1 with AZD3965 is undergoing clinical trials. We aimed to investigate nutrient fluxes associated with MCT1 inhibition by AZD3965 to identify possible biomarkers of drug action. We synthesized an ^18^F-labeled lactate analogue, [^18^F]-*S*-fluorolactate ([^18^F]-*S*-FL), that was used alongside [^18^F]fluorodeoxyglucose ([^18^F]FDG), and ^13^C-labeled glucose and lactate, to investigate the modulation of metabolism with AZD3965 in diffuse large B-cell lymphoma models in NOD/SCID mice. Comparative analysis of glucose and lactate-based probes showed a preference for glycolytic metabolism in vitro, whereas in vivo, both glucose and lactate were used as metabolic fuel. While intratumoral L-[1-^13^C]lactate and [^18^F]-*S*-FL were unchanged or lower at early (5 or 30 min) timepoints, these variables were higher compared to vehicle controls at 4 h following treatment with AZD3965, which indicates that inhibition of MCT1-mediated lactate import is reversed over time. Nonetheless, AZD3965 treatment impaired DLBCL tumor growth in mice. This was hypothesized to be a consequence of metabolic strain, as AZD3965 treatment showed a reduction in glycolytic intermediates and inhibition of the TCA cycle likely due to downregulated PDH activity. Glucose ([^18^F]FDG and D-[^13^C_6_]glucose) and lactate-based probes ([^18^F]-*S*-FL and L-[1-^13^C]lactate) can be successfully used as biomarkers for AZD3965 treatment.

## 1. Introduction

The monocarboxylate transporter 1 (MCT1) is a proton and monocarboxylate symporter that in humans is encoded by the SLC16A1 gene. MCT1, together with MCT4 (encoded by SLC16A3 gene), has been extensively studied for their roles in the cell membrane’s transport of monocarboxylates including lactate, pyruvate, and ketone-bodies [1]. Differential regulation of the two transporters by MYC (MCT1) and HIF-1 (MCT4), as well as through the cell membrane trafficking function of CD147/BASIGIN has been reported in cancer [2,3,4]. Selective inhibition of the MCT1 protein is being investigated for cancer therapy. Of the inhibitors, AZD3965 is the most advanced [5,6,7]. When tumors express both MCT1 and MCT4, as is the case in small cell lung cancer, MCT4 expression appears to be a negative prognostic factor for sensitivity to MCT1 inhibition [5]. This finding suggested that MCT1 inhibitor therapy will be most effective in cell lineages or tumor subpopulations expressing MCT1 but not MCT4. AZD3965 is currently undergoing a Cancer Research UK Centre for Drug Development sponsored Phase I trial in patients with advanced cancers (ClinicalTrials.gov identifier: NCT01791595). There is a need to identify clinically relevant biomarkers to monitor response to such inhibitors and future combination therapies.

Human diffuse large B-cell lymphoma (DLBCL) is the most frequent non-Hodgkin’s lymphoma subtype (~35%) and the most common lymphoid malignancy in adult patients. Incidence has doubled over that past decade highlighting the need for improved therapies [8], while only 60% of patients achieve long term remission after R-CHOP therapy. MYC aberrations, alone or concurrently with BCL2 aberrations, have been associated with inferior survival in DLBCL [9], warranting a study of MYC target gene products including MCT1 for therapy [10]. The purpose of this work was to investigate nutrient fluxes associated with MCT1 inhibition by AZD3965 in DLBCL mouse models, to identify possible biomarkers of drug action. To do this, we synthesized an ^18^F-labeled lactate analogue, [^18^F]-*S*-fluorolactate ([^18^F]-*S*-FL), that was used, alongside ^13^C-labeled glucose and lactate, and [^18^F] fluorodeoxyglucose ([^18^F]FDG) to determine the fate of lactate and its potential as a biomarker.

## 2. Results

### 2.1. Relevance of DLBCL as a Model for MCT1 Inhibition

**Figure 1:**
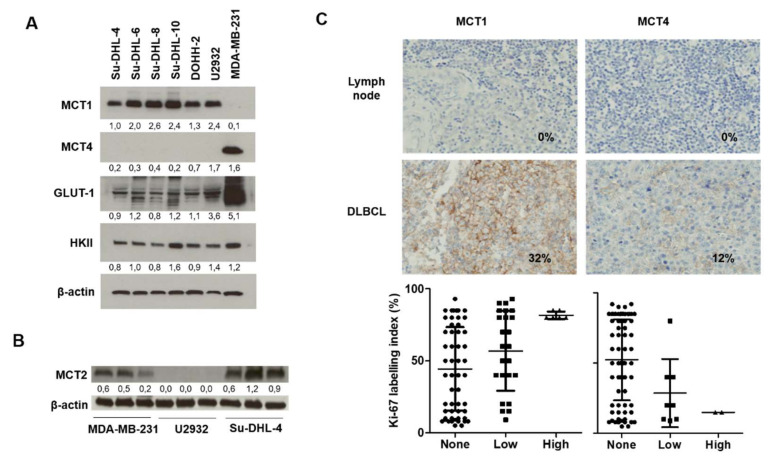
The monocarboxylate transporter MCT1 is expressed in diffuse large B cell lymphoma cell lines. (**A**) Expression of monocarboxylate transporters MCT1 and MCT4, glucose transporter GLUT-1 and hexokinase II in six diffuse large B cell lymphoma cell lines and the MDA-MB-231 cell line that is known to be deficient in MCT1 expression. (**B**) Expression of monocarboxylate transporter MCT2 in MDA-MB-231, U2932 and Su-DHL-4 cell lines. Values represent the ratio of protein to β-actin, which was used as a loading control; uncropped blots from (**A**) and (**B**) are shown in Figure S2. (**C**) Typical immunostains of MCT1 and MCT4 in uninvolved lymph nodes and stage I/II DLBCL (images above) and the relationship between MCT1/4 expression and Ki67 in stage I/II DLBCL (graphs below).

The expression of the lactate carriers MCT1 and MCT4, as well as glucose uptake enzymes, were evaluated in DLBCL cell lines and tumors (Figure 1). All human DLBCL cell lines studied (U2932, DOHH-2, Su-DHL-4, Su-DHL-6, Su-DHL-8, and Su-DHL-10) showed prominent expression of MCT1 protein and no MCT4 protein expression (Figure 1A). As a control, we used the MDA-MB-231 breast cancer cell line, which was deficient in MCT1 but proficient in MCT4 expression. All the cell lines expressed the glucose transporter GLUT-1 (highest in MDA-MB-231 cells) and hexokinase HKII, required for [^18^F]FDG cellular uptake and retention. We also investigated MCT2 expression—as it can also transport lactate and other monocarboxylates—in MDA-MB-231, U2932 and Su-DHL-4 cell lines and found that only U2932 is MCT2-negative (Figure 1B). The expression of MCT1 was also confirmed in DLBCL patient tissue microarray (TMA) samples (Figure 1C, Figure S1). Uninvolved lymph nodes lacked expression of either MCT1 or MCT4, while a proportion of (stage I/II DLBCL) tumors showed positive membrane expression. Notably, protein expression on the TMA samples was heterogeneous making accurate quantification difficult. Irrespective of this, a larger proportion of tumors were positive for MCT1 (low and high combined) than for MCT4 (Figure 1C). Furthermore, all patient samples classed as ‘MCT1-high’ (with scores between 101 and 300) had also high Ki67, while samples classed as ‘MCT4-high’ (with scores between 101 and 300) had low Ki67 expression. Ki67 expression was variable in MCT1 or MCT4 null (0 score) or low (0–100 score) tumors. Thus, DLBCLs appear suitable for AZD3965 therapy. To progress to in vitro evaluation of nutrient utilization, we chose two MCT1-positive cell lines, U2932 and SuDHL-4, and the MCT1-negative cell line, MDA-MB-231, for control purposes.

### 2.2. DLBCL Tumor Models Use Predominantly Glucose In Vitro

**Figure 2:**
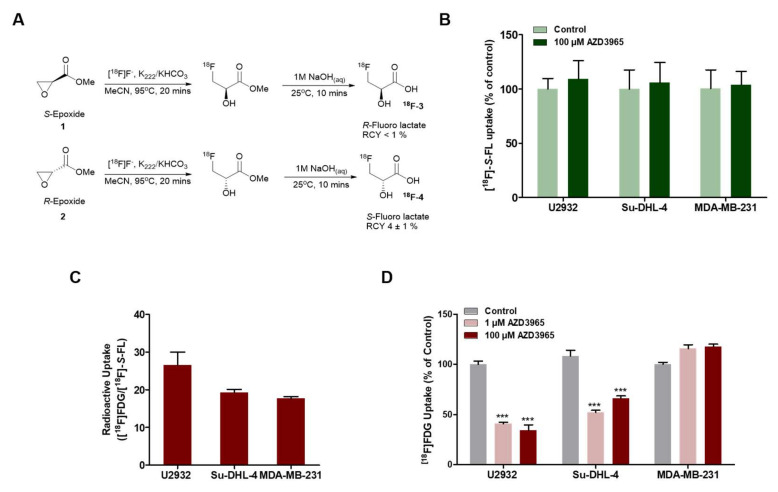
DLBCL cells predominantly utilize glucose in vitro. (**A**) Synthetic schemes for [^18^F]-*R*-fluorolactate and [^18^F]-*S*-fluorolactate ([^18^F]-*S*-FL). (**B**) Uptake of [^18^F]-*S*-FL (1h) in U2932, Su-DHL-4 and MDA-MB-231 cell lines in response to AZD3965 treatment (100 μM, 4h) (**C**) Comparative radioactive uptake of [^18^F]-*S*-FL and [^18^F]FDG (1h) expressed as the ratio of [^18^F]FDG relative to mean [^18^F]-*S*-FL. (**D**) Uptake of [^18^F]FDG (1h) following treatment with 1 or 100 mM of AZD3965 (4 h). All data are average ± SEM, expressed as percentage of control or as percentage of injected dose, normalized to protein content (%ID/mg) (*n* = 6–12). *** *p ≤* 0.005.

To investigate the metabolic phenotype of these models in vitro, we synthesized an ^18^F-labeled lactate analogue, [^18^F]*S*-fluorolactate ([^18^F]-*S*-FL) (Figure 2A). The naturally occurring form of lactate is the (*S*) or (*L*)-stereoisomer. When incorporating fluorine into the terminal carbon of the propyl chain in lactate, the nomenclature inverts, as the molecular weight of fluorine is higher than that of oxygen. Due to the inversion of the nomenclature, it is unclear that a difference in cellular uptake exists between the *R* and *D*-stereoisomer, analogous to *L*-lactate stereoisomer and the [^18^F]-*S*-fluorolactate. In any case, only the [^18^F]-*S*-fluorolactate could be synthesized in sufficient yield from stereochemically pure epoxides (Figure S3A,B) and the racemic mixture [^18^F]-R*S*-FL is taken up by cancer cells (Van Hée et al. 2017); [^18^F]-*S*-FL is but the enantiomeric form of the racemic mixture.

In vitro uptake of [^18^F]-*S*-FL in the MCT1-positive cell lines U2932 and Su-DHL-4 was not altered by treatment with 100 µM AZD3965 for 4 h (Figure 2B). This is not surprising considering that cultured cells are not expected to uptake lactate substantially in glucose-competent environments. Indeed, comparative radioactive incubation with [^18^F]FDG and [^18^F]-*S*-FL showed much higher intracellular accumulation of the former in all cell lines used (26.6, 19.3 and 17.7-fold in [^18^F]FDG-to [^18^F]-*S*-FL ratio of U2932, Su-DHL-4 and MDA-MB-231 cells, respectively—Figure 2C).

Cultured cells commonly rely on glycolysis for metabolism, where glucose is converted to pyruvate, which is then reduced to lactate via LDH and excreted from the cell [11]. The export of lactate under these conditions by MCT1 maintains intracellular pH and recycles NADH, both essential for sustaining glycolysis. In keeping with this, a more relevant indicator of the AZD3965 effect in vitro is the altered uptake of [^18^F]FDG. In vitro inhibition of MCT1 with 1 μM and 100 μM of AZD3965 for 4 h under the conditions of buffered culture pH (7.4) led to a drop in [^18^F]FDG uptake of 60 ± 1% and 65 ± 5% (*p* ≤ 0.0001) in U2932 cells and 48 ± 2% and 34 ± 2% (*p* ≤ 0.0001) in Su-DHL-4 cells (Figure 2D). This may be due to reduced cell viability and/or feedback inhibition of glycolysis consequent to intracellular accumulation of lactate, a phenomenon that has been reported in the literature following treatment with AZD3965 [6,12]; prolonged incubation (24 h) also showed a reduction of [^18^F]FDG uptake (Figure S4A). Treatment of MDA-MB-231 cells with AZD3465 neither altered [^18^F]-*S*-FL (Figure 2B) nor [^18^F]FDG (Figure 2D) uptake, in line with the absence of MCT1 in this cell line (Figure 1A) and the lack of selectivity of AZD3465 for MCT4 [6]. Cell viability of U2932 was also reduced to a larger extent than that of MDA-MB-231 cells in the presence of AZD3965 (IC50 of 110.7 and 230.1 nM, respectively; Figure S4B).

### 2.3. Lactate and Glucose are Used as Metabolic Fuels in DLBCL Xenografts

**Figure 3:**
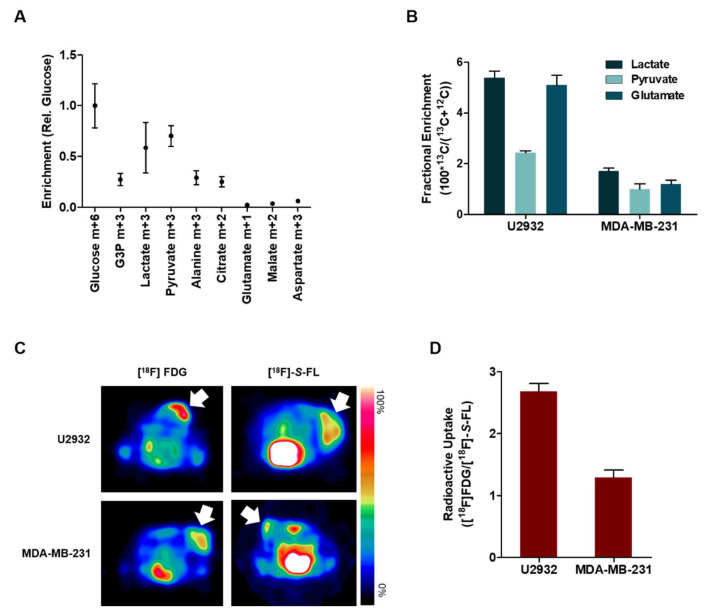
DLBCL tumor models utilize lactate as metabolic fuel. (**A**) Tissue enrichment of glucose m+6, glyceraldehyde-3-phosphate (G3P) m+3, lactate m+3, pyruvate m+3, alanine m+3, citrate m+2, glutamate m+1, malate m+2 and aspartate m+3 in U2932 tumor-bearing mice infused with D-[^13^C_6_]Glucose normalized to glucose enrichment. (**B**) Fractional enrichment of lactate, pyruvate and glutamate in U2932 and MDA-MB-231-tumor bearing mice infused with L-[1-^13^C]lactate. (**C**) Representative axial PET images of U2932 and MDA-MB-231 xenografts (**C**) and comparative radioactive uptake of [^18^F]-*S*-FL (30 min) and [^18^F]FDG (1 h) in both tumor models (**D**), expressed as the ratio of [^18^F]FDG relative to mean [^18^F]-*S*-FL. All data are average ± SEM (*n* = 4).

To investigate whether the same glycolytic phenotype is maintained in vivo, we infused tumor-bearing mice with D-[^13^C_6_]glucose. Metabolites extracted from U2932 tumors showed enriched downstream glycolytic intermediate glyceraldehyde-3-phosphate m+3 (G3P) relative to glucose m+6 (Figure 3A), indicative of glycolytic activity. Interestingly, lactate, pyruvate and alanine had excess labeling relative to G3P; a high lactate-to-G3P ratio is a surrogate for utilization of these 3-carbon intermediates from circulation [13]. Thus, the high lactate-to-G3P ratio (2.1 ratio) suggests the systemic conversion of labeled glucose into lactate and its subsequent import from plasma.

In a similar protocol, the infusion of L-[1-^13^C]lactate showed intracellular accumulation of enriched lactate in U2932 tumors (5.4%) (Figure 3B), also consistent with lactate import from circulation. Furthermore, the presence of ^13^C-enriched pyruvate, as well as glutamate (2.4% and 5.1%, respectively) indicates the exchange of labeled lactate with these intermediates. MDA-MB-231 tumors showed less accumulation of enriched metabolites (1.7%, 1.0% and 1.2% of lactate, pyruvate and glutamate, respectively). Comparative radioactive uptake of [^18^F]FDG and [^18^F]-*S*-FL in tumor-bearing mice showed that both tracers were taken up (Figure 3C), with [^18^F]FDG uptake being higher than [^18^F]-*S*-FL (2.7 and 1.3-fold [^18^F]FDG to [^18^F]-*S*-FL ratio in U2932 and MDA-MB-231 xenografts, respectively) (Figure 3D).

### 2.4. Pharmacodynamics of Lactate Transport Inhibition by AZD3965

**Figure 4:**
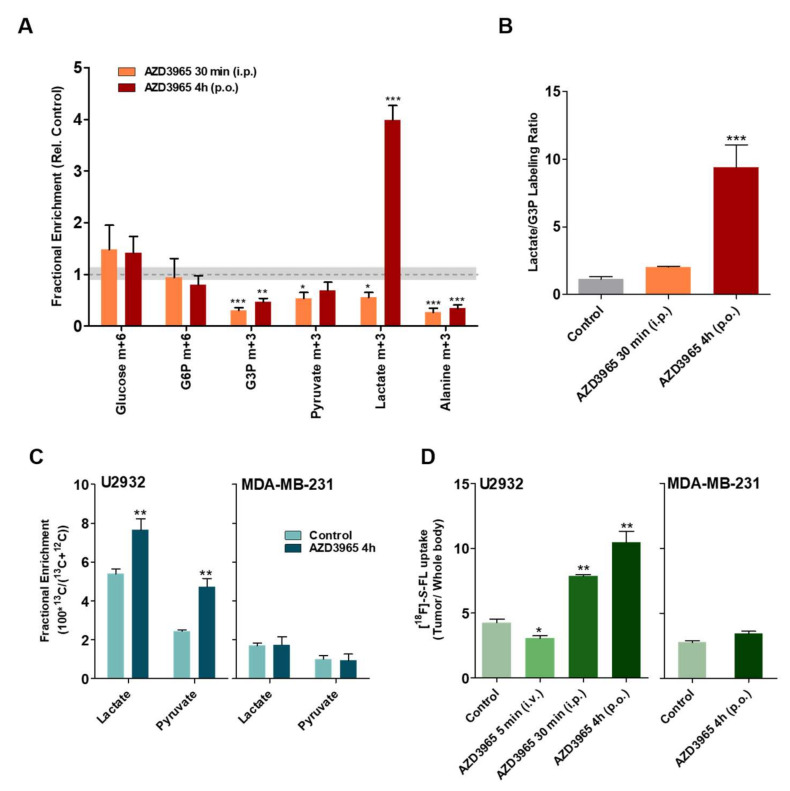
AZD3965-mediated inhibition of lactate import is reversed in DLBCL tumors. (**A**) Tissue enrichment of glucose m+6, glucose 6-phosphate (G6P) m+6, glyceraldehyde-3-phosphate (G3P) m+3, pyruvate m+3, lactate m+3 and alanine m+3 in U2932-bearing mice infused with D-[^13^C_6_]glucose following treatment with AZD3965 (100 mg/kg) for 30 min (i.p.) or 4 h (p.o.). Data are expressed as fractional enrichment relative to control (vehicle), indicated by the dotted line (average) and shaded bar (SEM). (**B**) Lactate to glyceraldehyde-3-phosphate (G3P) labeling ratios of tumors from (A). (**C**) Fractional enrichment of lactate and pyruvate in U2932 (left) and MDA-MB-231(right)-tumor bearing mice infused with L-[1-^13^C]lactate following treatment with AZD3965 (100 mg/kg) for 4 h. (**D**) Radioactive uptake of [^18^F]-*S*-FL after treatment with 100 mg/kg of AZD3965 for 5 min (i.v.), 30 min (i.p.) in U2932 tumors and 4 h (p.o.) in both U2932 and MDA-MB-231 tumors, normalized to whole body uptake. All data are average ± SEM (*n* = 4 ). * *p* ≤ 0.05, ** *p* ≤ 0.01 and *** *p* ≤ 0.005.

To evaluate the impact of MCT1 inhibition in lactate transport, we traced cellular flux of metabolites by infusing tumors with exogenous D-[^13^C_6_]glucose, L-[1-^13^C]lactate and [^18^F]-*S*-FL (Figure 4) during AZD3965 treatment. In mice infused with D-[^13^C_6_]glucose, AZD3965 treatment resulted in a small reduction of some glycolytic intermediates, with the most profound effect on the concentration of intracellular enriched lactate: whereas treatment for 30 min diminished lactate m+3 (*p* ≤ 0.05), a dramatic increase (4-fold, *p* ≤ 0.005) was measured at 4 h of treatment (Figure 4A). Combined with lower G3P (54% drop relative to control at 4 h p.o., *p* ≤ 0.005), AZD3965 treatment resulted in a 10-fold increase in the lactate-to-G3P ratio (Figure 4B); considering that high lactate-to-G3P ratio is an indicator for lactate uptake and utilization distal to glycolysis [13], this increase suggests that lactate import is not inhibited at 4 h of treatment with AZD3965. Indeed, infusion of L-[1-^13^C]lactate also showed a 40% increase (*p* ≤ 0.005; Figure 4C-left) of enriched lactate in the U2932 tumors after 4 h treatment accompanied by a non-significant reduction of lactate in plasma (Figure S5A).

The presence of enriched pyruvate—that also increased following AZD3965 treatment (*p* ≤ 0.005; Figure 4C) suggests that the imported lactate was converted into pyruvate in the presence of LDH (Figure S5B). The control tumor, MDA-MB-231, showed no significant changes (Figure 4C-right). Uptake of [^18^F]-*S*-FL by U2932 tumors followed a similar trend: whereas treatment with AZD3965 for 5 min (i.v.) led to lower uptake (*p* ≤ 0.05, Figure 4D), an almost 2-fold increase (88%, *p* ≤ 0.01) in [^18^F]-*S*-FL content was seen at 4 h (p.o.) relative to the control group; this is true for both tumor uptake normalized to the whole body (Figure 4D) and for NUV_30_ values (Figure S6A) while the uptake in muscle did not change over time (Figure S6A). In MCT1-negative MDA-MB-231 tumors, [^18^F]-*S*-FL uptake was not affected by AZD3965 treatment (Figure 4D-right). This seems to indicate that AZD3965 pharmacodynamics are transient in the MCT1-positive xenografts, and that inhibition of lactate influx at 5 min is reversed within 4 h. Relatively low levels of radioactive uptake were seen in bone after 1 h of [^18^F]-*S*-FL administration, indicating a low level of defluorination of the radiotracer (Figure S6).

### 2.5. AZD3965 Impairs TCA Cycle and Inhibits Tumor Growth in DLBCL

**Figure 5:**
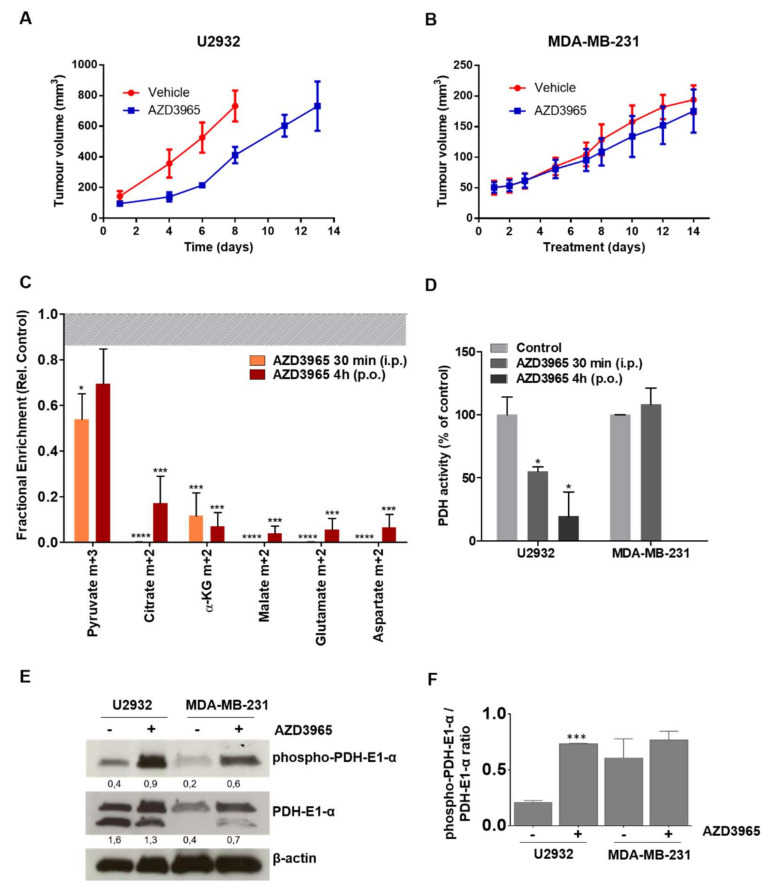
AZD3965 treatment inhibits tumor growth, the TCA cycle and PDH activity. (**A**) Anti-tumor activity of AZD3965 (100 mg/kg p.o. twice daily, *n* = 6–8) against U2932 and (**B**) MDA-MB-231 xenografts. (**C**) TCA cycle metabolite enrichment (pyruvate m+3, citrate m+2, α-ketoglutarate (α-KG) m+2, malate m+2, glutamate m+2, aspartate m+2) in U2932-tumor-bearing mice infused with D-[^13^C_6_]glucose following treatment with AZD3965 (100 mg/kg) for 30 min (i.p.) or 4 h (p.o.). Data are expressed as fractional enrichment relative to control (vehicle), indicated by the dotted line (average) and shaded bar (SEM). (**D**) Analysis of PDH activity by Enzyme-Linked Immunosorbent Assay (ELISA) in U2932 and MDA-MB-231 tumors excised from mice treated with AZD3965 (100 mg/kg) or vehicle (control) for 30 min or 4 h. (**E**) Representative blots and (**F**) respective quantification (*n* = 3) of the expression of phospho-PDHE1-α and PDHE1-α in tumors excised from U2932 and MDA-MB-231-tumor bearing mice treated with AZD3965 (100 mg/kg) or vehicle (control) for 4 h; values represent ratio of protein to β-actin, which was used as loading control; uncropped blots are shown in Figure S8. All data are average ± SEM (*n* = 4–6). * *p* ≤ 0.05, *** *p* ≤ 0.005 and **** *p* ≤ 0.001.

We investigated the effect of AZD3965 in mice and found that treatment at a pharmacologically relevant dose (100 mg/kg BID by oral gavage) [6,14,15] delayed tumor growth in U2932 xenografts but lacked efficacy in MDA-MB-231 xenografts (Figure 5A,B). The treatment regimen used did not affect the mouse weight and no adverse effect. In a tumor model that utilizes lactate as fuel, we were interested in investigating the mechanism through which the accumulation of intracellular lactate following AZD3965 treatment disrupts tumor growth.

Infusion of D-[^13^C_6_]glucose showed a marked reduction of labeled TCA cycle intermediates at both 30 min (*p* ≤ 0.001) and 4 h (*p* ≤ 0.005) of treatment (Figure 5C). Notably, this was not only the product of reduced glycolysis since the levels of pyruvate m+3 are only moderately affected. The conversion of pyruvate into acetyl-coA and shuttling to the TCA cycle is regulated by the pyruvate dehydrogenase (PDH) complex. PDH activity is inhibited through phosphorylation by pyruvate dehydrogenase kinase (PDK) in response to substrate accumulation or pH variations [16,17]. The intracellular accumulation of lactate and consequent acidification may induce inactivation of the PDH complex, which prevents the flux of pyruvate into the TCA cycle. Indeed, a variation of pH in U2932 tumor averaged over the entire tumor of up to −4 units was measured by ^31^P MRS in the first 1 h of AZD3965 treatment, which was reversed at 4 h (Figure S7). Furthermore, an ELISA assay showed a marked reduction of PDH activity in U2932 tumors after treatment (48% at 30 min and 58% at 4 h, Figure 5D), which was accompanied by an increase in the expression of phospho-PDH in this tumor model (*p* ≤ 0.005, Figure 5E), but not in MDA-MB-231 tumors (Figure 5D,E).

## 3. Discussion

AZD3965 represents the first selective MCT1 inhibitor to reach clinical trials, and biomarkers that can accurately predict treatment efficacy are of critical importance. The mechanism of AZD3965-mediated toxicity has been reported to be through intracellular lactate accumulation, which disrupts important cellular functions such as glycolysis, inducing cell death [6]. Cancer metabolism is, however, a complex landscape: the inter- and intra-tumoral fate of lactate can be highly heterogeneous, possibly affecting treatment pharmacodynamics. In this work, we developed an ^18^F-labeled lactate analogue and used it alongside L-[1-^13^C]lactate, D-[^13^C_6_]glucose and [^18^F]FDG to investigate the altered nutrient flux of tumors in the presence of the MCT1 inhibitor AZD3965: we present evidence that lactate is consumed by the DLBCL tumor model used, and, perhaps consequentially, AZD3965-mediated inhibition of lactate influx is reversed within 4 h of treatment.

Lactate transport across the cell membrane is largely mediated by the MCT family, with the MCT1 and MCT4 being particularly important in cancer metabolism [18]. Whereas MCT1 can transport lactate bi-directionally, MCT4 is mainly responsible for its efflux [19]; MCT4 expression has been identified as a potential resistance mechanism to MCT1 abrogation [5] and can hinder the efficacy of AZD3965, an MCT1-selective inhibitor that has no reported effect on MCT4 [6]. Thus, we chose DLBCL models with high expression of MCT1 but negative for MCT4 and considered MDA-MB-231—a breast cancer model, negative for MCT1 but positive for MCT4—a suitable insensitive control. Furthermore, as AZD3965 also has activity against MCT2, we chose a negative (U2932) and a positive (Su-DHL-4) model to appreciate any possible confounding effects.

Most tumor cells are highly glycolytic—indeed, increased glucose metabolism underpins the success of [^18^F]FDG as an imaging agent—and produce lactate as a metabolic by-product. Co-efflux of lactate and H^+^ through MCT1 (and MCT4) allows intracellular pH to be maintained and recycles NADH which can be used for glycolysis, thus sustaining it [20]. The high glycolytic phenotype of our tumor cells in vitro was evident by the substantially higher uptake of [^18^F]FDG compared to [^18^F]-*S*-FL. Furthermore, the relationship between MCT1 and glycolysis was exemplified in our data, where cells in culture showed a decrease in [^18^F]FDG uptake shortly after AZD3965 treatment, suggesting feedback inhibition of glycolysis. Consistent with the glycolytic phenotype of the models in vitro, [^18^F]-*S*-FL uptake was poor and did not change upon MCT1 inhibition.

In vivo, however, a different phenotype was shown: U2932 tumors infused with D-[^13^C_6_]glucose showed excess enrichment of lactate, pyruvate and alanine relative to the glycolytic intermediate G3P, which indicates uptake and utilization of these 3-carbon intermediates from circulation, distal to glycolysis [13]. Infusion with L-[1-^13^C]lactate in these tumors further confirmed uptake of enriched lactate and exchange with glutamate and pyruvate, indicating its use as a fuel [21]. Moreover, the ratio of uptake of [^18^F]FDG/[^18^F]-*S*-FL in xenografts is lower than that in culture, and although this is not informative of absolute ratios of the utilization of lactate and glucose, it suggests that both substrates are used concomitantly. It is not uncommon for cancer cells to exhibit different behaviors in vitro and in vivo as these environments do not pose the same metabolic challenges. Indeed, MCT1 has been shown to, within the same tumor model, sustain a glycolytic phenotype in vitro by exporting lactate, while importing it from the circulation to be used as a fuel in vivo [13]. This is part of new research that brought to light that lactate is not only a waste product of glycolysis but, is often used as a metabolic fuel. Sonveaux and colleagues [22], for example, have shown that while hypoxic tumor cells primarily rely on glucose for energy production, the lactate produced during glycolysis is used as a substrate that fuels oxidative metabolism in oxygenated cells. Since then, similar multi-compartment models where lactate is exchanged between different cancer-associated cells in metabolic synergy have been proposed [23]. Although it is difficult to resolve such delicate differences between lactate production/utilization when considering the whole tumor landscape, other studies have reported the net flux of lactate consumption by tumors [14,21,24] including DLBCL [25]. Our data suggest that lactate is not only imported from the circulation but is also used to supply the TCA cycle. The use of lactate as a fuel does not, however, preclude glycolysis: the presence of glycolytic intermediates indicates that glucose and lactate are used concomitantly. This phenomenon has been reported in the literature and represents a metabolic adaptation that allows tumors to survive under variable metabolic conditions [13,21,26]. Nonetheless, our tumor model showed low dependence on glucose uptake and glycolysis, as inhibition of lactate transport with AZD3965 showed little impact on the enrichment of glycolytic intermediates glucose m+6 and G3P m+3 following infusion with D-[^13^C_6_]glucose. Interestingly, while enriched lactate accumulated less in the tumors at 30 min of treatment, compatible with inhibition of lactate transport, a dramatic increase of lactate-to-G3P enrichment ratio was seen at 4 h, and together with a reduction of labeled lactate in plasma, this rather suggests increased import (and utilization) of lactate from circulation. This is seemingly in contradiction with the expected AZD3965-mediated inhibition of lactate influx [5,6,27], but these results were corroborated by the higher uptake of both [^18^F]-*S*-FL and L-[1-^13^C]lactate following 4 h treatment with AZD3965.

Due to its role as both by-product of glycolysis and metabolic fuel, transport of lactate will be highly heterogeneous between and within tumors; whether it is imported or exported will depend on transient metabolic needs, substrate availability or proton gradients. Thus, investigating lactate transport in vivo is not a straightforward task. Most studies to date report an increase in intracellular lactate following AZD3965 treatment, but the caveat of measuring an endogenous metabolite is the inability to distinguish its origin, which severely restricts the capacity to infer about its transport. Indeed, measurements of intracellular endogenous lactate often do not correlate with MCT1 expression [12] or are seemingly reversible over time [6]. Exogenous labeled lactate analogues are therefore better suited to investigate the flux of lactate in and out of cells. In a related study where an ^18^F-labeled lactate analogue ([^18^F]FLac) [27] is also evaluated as a biomarker for AZD3965, a decrease in uptake is seen during acute treatment (10 min)—similarly to the decrease in [^18^F]-*S*-FL reported in this work after 5 min of treatment—but prolonged treatment was not studied. Put together, our data seems to indicate that AZD3965 pharmacodynamics are reversible over time, and that inhibition of MCT1-mediated lactate influx is reversed at 4 h. The physiological context could have also played a role. We noted that AZD3965 induced intracellular acidification within 1 h of injection which was reversed by 4 h. In the attempt to normalize intracellular pH, transient build-up of protons in the extracellular space through different transporters [28] could lead to a proton gradient that increases [^18^F]-*S*-FL uptake. Similar phenomena have been previously reported: Guan et al. [7] observed an ‘overshoot’ in L-lactate uptake beyond baseline levels after prolonged treatment with AZD3965, which was attributed to the reversibility of MCT1 inhibition. In another study [29], a different MCT1 inhibitor, syrosingopine, was also found to increase uptake of L-[^3^H]lactate, suggesting that, over time, lactate export is more efficiently inhibited than import.

It is interesting to consider the implications of such a phenotype: if intracellular accumulation of lactate induces metabolic catastrophe in glycolytic cells, how does it impact net bioenergetics—and growth—in cells that oxidize lactate as a fuel? Remarkably, and despite its unexpected effect on the transport of lactate, AZD3965 considerably delayed tumor growth in our sensitive model. We propose that this is due to treatment-induced disruption of glycolysis (glycolytic intermediates) and the TCA cycle. For DLBCL tumors that utilize lactate for TCA metabolism, inhibition of PDH complex—the gate-keeper enzymes that shuttle pyruvate into mitochondrial acetyl-CoA to enter the TCA cycle—could be detrimental for growth, as TCA metabolism provides both energy and nucleotide building blocks via aspartate [30,31,32], one of the metabolites that decreased after AZD3965 treatment. What is less clear is how AZD3965 inhibits PDH; it is plausible that lactate accumulation induces intracellular acidification to inhibit the acid-sensitive PDH. The low magnitude of intracellular pH reduction (~0.4 units averaged over the tumor) and compartmental nature of PDH (within mitochondria instead of cytosol) makes it difficult to assign the loss of PDH activity entirely to low pH.

Interestingly, whereas some groups show similar reductions in TCA cycle intermediates and bioenergetics following MCT1 inhibition [2], others report seemingly contradictory results: Beloueche-Babari et al. showed glucose routing towards mitochondrial metabolism through increased PDH flux and consequently improved energy production after AZD3965 treatment [14]. This apparent metabolic adaptation provides support for survival pathways which can lead to drug resistance [33]. Thus, while the impact of AZD3965 treatment in intracellular lactate accumulation or bioenergetics is highly dependent on the tumor’s phenotype, reduced TCA cycle activity may represent more robust biomarkers of treatment efficacy.

In this work, the use of imaging paired with labeled exogenous fuels clearly showed the uptake and utilization of lactate, perhaps concomitantly to glycolysis. Whereas it is difficult to evaluate whether both metabolic pathways occur within the same cell or what proportion of glucose/lactate is used to fuel metabolism at any point, this work joins a body of literature that incentivizes revision of traditional metabolism concepts such as the Warburg effect. The complexity of tumor metabolism, including intra and inter-tumor metabolic heterogeneity, cell’s microenvironment and/or fuel availability should be considered when developing new therapeutics and imaging biomarkers. Nonetheless, we showed that [^18^F]-*S*-FL—but also D-[^13^C_6_]glucose and L-[1-^13^C]lactate—can be used as biomarkers to evaluate AZD3965 treatment response.

## 4. Materials and Methods

### 4.1. Cell Lines and Drugs

Human Diffuse Large B cell Lymphoma (DLBCL) cells Su-DHL-4, Su-DHL-6, Su-DHL-8 and Su-DHL-10 were obtained from Dr Li Jia (Barts Cancer Institute, London, UK); DOHH-2 and U2932 were purchased from Deutsche Sammlung von Mikroorganismen und Zellkulturen GmBH (Braunschweig, Germany). Luciferase-expressing human breast cancer cell line MDA-MB-231 was purchased from Perkin Elmer (Waltham, MA, USA). All cells were passaged in our laboratory for fewer than 6 months on receipt and were tested mycoplasma free. Experiments were performed using cells under 10 passages. AZD3965 was obtained as a gift from AstraZeneca (Macclesfield, UK); MCT1 inhibitor α-Cyano-4-hydroxycinnamic acid (CHC) was purchased from Sigma-Aldrich (Dorset, UK).

### 4.2. Synthesis of [^18^F]-S-FL

A detailed experimental for the radiosynthesis of [^18^F]-*S*-FL is reported in the Supplementary Methods. In brief, dry [^18^F]fluoride in MeCN was added to the methyl-2R-glycidate precursor and heated to 95 °C for 20 min. The reaction mixture was purified by SPE extraction and the radiolabeled intermediate was subsequently hydrolyzed under basic conditions (NaOH, 1M) to give [^18^F]-*S*-FL in a radiochemical yield of 4 ± 1 % (n > 15).

### 4.3. Western Blot

Western blot was performed using standard techniques. For detailed methodology, see Supplementary Methods; for original, uncropped blots, see Figure S2, S8 and S10 from Supplementary Materials file.

### 4.4. Immunohistochemistry

The commercial panels of DLBCL tissue microarray (TMA) LY1002 and normal lymph node TMA BNC20011 section were obtained from Biomax Inc (Rockville, MD, USA). They were provided with a Hematoxylin & Eosin stained virtual image that was used to assess the morphology. Immunohistochemistry was performed using standard techniques. For detailed methodology, see Supplementary Methods.

### 4.5. In Vitro [^18^F]FDG and [^18^F]-S-FL Uptake

For uptake studies, cells were plated at a density of 2 × 10^6^ cells/well in 6-well plates (*n* = 6) and allowed to recover for 24 h. To permit sensitive [^18^F]FDG detection, all cells were grown in 2.5 mM glucose media overnight; on the day of the assay, cells were pre-incubated with 1 or 100 µM of AZD3965 for 4 h or 1, 10 or 100 nM of AZD3965 (in the presence or absence of 10 mM lactate); or 5 mM CHC for 24 h in 2.5 mM glucose media. pH of the media was adjusted to 7.4 to avoid confounding effects of pH. Prior to incubation with radioactivity, the medium was discarded and cells were quickly washed three times with PBS followed by incubation with 0.74 MBq/well of [^18^F]FDG or [^18^F]-*S*-FL in 2.5 mM glucose-containing media for 60 min. Cells were incubated in media containing 2 g/L sodium bicarbonate in the presence of CO_2_ at 37 °C. After incubation, radioactivity was discarded and cells were washed with 1 mL of ice-cold PBS three times with centrifuging (13,000 rpm for 3 min) for the suspension cell cultures (all DLBCL cells). Cells were lysed with 1 mL of RIPA buffer (Sigma-Aldrich) for 15 min on ice and radioactivity of the lysate was measured by γ-counting. Counts/min data were expressed as a percentage of the incubated dose (ID) of radioactivity normalized to the total cellular protein concentration of the sample, determined using Pierce^®^ BCA assay (Thermo Fisher Scientific, Loughborough, UK), i.e., %ID/mg protein or as ratios to control. Variations in the experimental protocol used due to the different (adherent/non-adherent) phenotype of the cell lines are captured in the relative ratio of [^18^F]FDG to [^18^F]-*S*-FL.

### 4.6. Animal Experiments

All animal experiments were performed by licensed investigators under Project License 7,008,651 approved on 28 July 2015 by the Animal Welfare Ethical Review Body (AWERB), and in accordance with the National (UK Home Office) Guidance on the Operation of the Animal (Scientific Procedures) Act 1986 (HMSO, London, UK, 1990) and within the guidelines set out by the UK National Cancer Research Institute Committee on Welfare of Animals in Cancer Research [34] and ARRIVE guidelines. The in vivo models were established by injecting U2932 cells (5 × 10^6^) subcutaneously on the back of female NOD/SCID mice (Charles River UK Ltd., Margate, UK) or MDA-MB-231 cells (5 × 10^6^) on the back of female nu/nu-BALB/c athymic nude mice (Charles River). Inoculations were carried out under 2% isoflurane/O_2_ anesthesia 7 days after animal arrival. All mice were 10–16 weeks of age and of similar weight (20 ± 3g) and kept under standard conditions in individually ventilated cages (maximum of 6 animals per cage) in a designated SPF laboratory in the containment room in the animal facility.

### 4.7. In Vivo Imaging

To evaluate tumor uptake of [^18^F]FDG and [^18^F]-*S*-FL, PET imaging was performed using standard techniques. For detailed methodology, see Supplementary Methods.

### 4.8. In Vivo D-[^13^C_6_]glucose and L-[1-^13^C]lactate Uptake Studies

Schema describing the protocol used to assess D-[^13^C_6_]glucose and L-[1-^13^C]lactate tumor is shown in Figure S9. Metabolites were analyzed as previously described for GC-MS analysis [14] or LC-MS analysis [15]. For detailed methodology, see Supplementary Methods.

### 4.9. Enzyme-Linked Immunosorbent Assay (ELISA)

Excised snap frozen tumor tissue samples were homogenized by grinding in a previously cooled mortar and pestle. Liquid nitrogen was added to ensure a powder was obtained and the temperature was kept low; subsequently, tissues were lysed in RIPA buffer (Sigma-Aldrich). PDH activity was determined by ELISA (Abcam, Cambridge, UK, Pyruvate Dehydrogenase (PDH) Enzyme Activity Microplate Assay Kit, ab109902) according to the manufacturer’s instructions.

### 4.10. Statistical Analysis

GraphPad Prism version 6.00 for Windows (GraphPad Software, La Jolla, San Diego, CA USA) was used for analysis. Data were expressed as mean ± SEM. Unpaired two-tailed Student’s *t*-test was used for statistical analysis of two data sets; analysis of variance (ANOVA) was used for >2 datasets. Differences between groups were considered significant if *p* ≤ 0.05.

## 5. Conclusions

In this work, we investigated nutrient flux in a DLBCL model through radioactive uptake of exogenously labeled fuels—[^18^F]-*S*-FL, synthesized in our group, together with [^18^F]FDG, L-[1-^13^C]lactate and D-[^13^C_6_]glucose following treatment with MCT1 inhibitor, AZD3965.

We have shown that while cells present a glycolytic phenotype in culture, in vivo, lactate was taken up and used in parallel to glucose utilization. Furthermore, treatment with AZD3965 increased tumor uptake of both L-[1-^13^C]lactate and [^18^F]-*S*-FL, suggesting that MCT1-mediated inhibition of lactate import is reversed over time. Nonetheless, AZD3965 reduced tumor growth in mice possibly due disruption of bioenergetics and the TCA cycle, as suggested by the reduction of TCA cycle intermediates paired with downregulation of PDH activity. Independently from drug action, we have shown that [^18^F]-*S*-FL—but also D-[^13^C_6_]glucose and L-[1-^13^C]lactate—can be used as biomarkers to evaluate AZD3965 treatment response.

## Supplementary Materials

The following are available online at https://www.mdpi.com/2072-6694/12/6/1703/s1, Figure S1: Validation of commercial antibodies for detection of MCT1 and MCT4 in tissue sections, Figure S2: Whole western blots corresponding to cropped bands in (**A**) Figure 1A and (**B**) Figure 1B; Figure S3: Characterization of [^18^F]-*S*-FL; Figure S4: In vitro effect of AZD3965 treatment; Figure S5: Concentration of lactate in plasma and LDH expression; Figure S6: In vivo uptake of [^18^F]-*S*-FL; Figure S7: pH variations caused by AZD3965 treatment; Figure S8: Whole western blots corresponding to cropped bands in Figure 5E; Figure S9: Scheme illustrating timing of in vivo experiments involving [^18^F]FDG, [^18^F]-*S*-FL, L-[1-^13^C]lactate and D-[^13^C_6_]glucose injections; Figure S10: Whole western blots corresponding to cropped bands in Figure S5B. Supplementary Methods describing: Synthesis of [^18^F]-*S*-FL, Western Blot, Immunohistochemistry, In Vivo Imaging and In Vivo D-[^13^C^6^]glucose and L-[1-^13^C]lactate Uptake Studies.
