# Supplementary material for: Tracing Nutrient Flux Following Monocarboxylate Transporter-1 Inhibition with AZD3965
Source: Cancers (Basel). 2020 Jun 26;12(6):1703. doi: 10.3390/cancers12061703 (PMC7352845; doi:10.3390/cancers12061703)
Supplement: Supplementary file 1 [file cancers-12-01703-s001.pdf]

Supplementary Material: Tracing Nutrient Flux Following Monocarboxylate Transporter-1 Inhibition with AZD3965

Marta Braga, Maciej Kaliszczak, Laurence Carroll, Zachary T. Schug, Kathrin Heinzmann, Nicoleta Baxan, Adrian Benito, Gabriel N. Valbuena, Stephen Stribbling, Alice Beckley, Gillian Mackay, Francesco Mauri, John Latigo, Chris Barnes, Hector Keun, Eyal Gottlieb and Eric O. Aboagye

1. Supplementary Methods

1.1. Synthesis of [^{18}F]-S-FL

[^{18}F]fluoride was produced by a cyclotron using the ^{18}O(p,n) ^{18}F nuclear reaction with 16.4 MeV proton irradiation of an enriched ^{18}O-H_2O target. Analytical reverse-phase HPLC was carried out on an Agilent 1200 instrument using a Phenomenex Gemini C18 column (150 mm × 4.6 mm) with a gradient of methanol containing 0.1 M tetrabutylammonium iodide and 0.1 M Na_2HPO_4 1.5 mL/min; a UV wavelength of 254 nm was used. Laura 3 software (LabLogic, Sheffield, UK) was used for processing all HPLC chromatograms. Methyl-2R-glycidate (5 µL, 0,06 µmol) was added to 300 µL of MeCN. Subsequently, [^{18}F]fluoride (containing kryptofix 2.2.2 (7 mg) and $KHCO_3$ (2 mg), dried azeotropically with MeCN and then re-dissolved in MeCN (300 µl)) was added and the reaction mixture was heated to 95 °C for 20 min. The reaction mixture was cooled to room temperature, before diluting with H_2O (20 mL), and passed through an alumina cartridge and tC18 light SPE cartridge (prepared with EtOH (2 mL) and H_2O (5 mL)) in series. The labeled compound was trapped on the tC18 SPE cartridge, which was then washed with H_2O (2 × 5 mL), before being eluted using EtOH (5 × 100 µL fractions). 1M NaOH (100 µL) was added and the mixture was allowed to sit for 10 min, before being neutralized with 1M HCl (~100 µL; pH was confirmed with pH paper). Analytical radio-HPLC was used to confirm the radiochemical purity of [^{18}F]-*S*-FL (rt = 8 min) (Figure S2), whilst attempts to measure the specific activity proved difficult due to the lack of a UV chromophore in the reference material, giving a non-representative peak height. The radiochemical yield was found to be 4 ± 1 % ($n > 15$).

1.2. Western Blot

Cells were washed 3 times with ice-cold PBS and lysed with RIPA buffer (Sigma-Aldrich, Dorset, UK) supplemented with 100X Pierce™ protease and phosphatase inhibitor cocktail (Thermo Fisher Scientific, Loughborough, UK) for 15 min on ice. Tumor samples were prepared by grinding in a previously cooled mortar and pestle. Liquid nitrogen was added to ensure a powder was obtained and that temperature was kept low. Samples were subsequently lysed with RIPA buffer (Sigma-Aldrich) supplemented with 100X Pierce™ protease and phosphatase inhibitor cocktail (Thermo Fisher Scientific) for 15 min on ice. Samples were centrifuged at 500 rpm for 5 min at 4 °C and the supernatant transferred to a new Eppendorf tube. Total protein concentration was quantified using the Pierce™ BCA protein assay kit (Thermo Fisher Scientific). Lysates were mixed with NuPage® LDS loading buffer and reducing agent (Invitrogen, Paisley, UK), and denatured at 70 °C for 10 min. Equal amounts of protein (30 µg) were resolved on 4–15% Mini-PROTEAN® TGX™ gels (Bio-Rad, Hemel Hempstead, UK) and separated by gel electrophoresis. The gels were then transferred to PVDF membranes (Trans-Blot Turbo Transfer Packs, Bio-Rad) using the Trans-Blot® Turbo™ System (Bio-rad). Membranes were blocked for 1 h in 5% milk in PBS containing 0.1% v/v Tween® 20 (Sigma-Aldrich) (PBST) and incubated with anti-MCT1 polyclonal antibody (anti-SLC16A1, Sigma-Aldrich) at 1/1000 dilution, anti-MCT4 polyclonal antibody (anti-SLC16A3, Sigma-Aldrich) at 1/500 dilution, anti-MCT2 monoclonal antibody (1/1000 dilution, SC-166925, Santa Cruz Biotechnology, Dallas, USA), anti-GLUT-1 (D3J3A) monoclonal antibody (12939, Cell Signalling Technology, Danvers, USA)

at 1/6000 dilution, anti-Hexokinase II (C64G5) monoclonal antibody (2867, Cell Signalling Technology) at 1/1000 dilution anti-phospho-PDHE1-α type I (Ser293) polyclonal antibody (ABS204, Millipore UK Ltd, Hertfordshire, UK) at 1/1000 (cell samples) and 1/500 (tumor samples) dilutions and anti-PDHE1-α polyclonal antibody (PA5-21536, Thermo Fisher Scientific) at 1/5000 dilution in 5% milk-TBST overnight at 4 °C. After washing with PBST, goat anti-rabbit Immunoglobin G (IgG) horseradish peroxidase (IgG-HRP) (1/2000 dilution, SC-2004, Santa Cruz Biotechnology) or goat anti-mouse lgG-HRP (1/2000 dilution, SC-2005, Santa Cruz Biotechnology) were incubated in 5% milk-PBST for 1 h at room temperature. Signals were detected using Amersham enhanced chemiluminescence (ECL) Plus Western Blotting Detection Reagent kit (GE Healthcare Life Sciences, Buckinghamshire, UK) and Amersham Hyper-film (GE Healthcare Life Sciences). Intensity of protein bands was normalized to β-actin (mouse anti-β-actin antibody (1/10,000 dilution, ab6276, Abcam, Cambridge, UK)) and analyzed using ImageJ version 1.44h.

1.3. Immunohistochemistry

The commercial panels of DLBCL tissue microarray (TMA) LY1002 and normal lymph node TMA BNC20011 section were obtained from Biomax Inc (Rockville, MD, USA). They were provided with a Hematoxylin & Eosin stained virtual image that was used to assess the morphology. Tissues were collected under the highest ethical standards with the donor being informed completely and with their consent; all human tissues were collected under Health Insurance Portability and Accountability Act-approved protocols (http://www.biomax.us/faq.php#q10). The TMA sections were de-paraffinized in xylene, rehydrated in graded alcohols and heated in a microwave oven at 900 W for 30 min in citrate buffer at pH 6.0. Endogenous peroxidase activity was suppressed before immunostaining with Powerblock reagent (Biogenex, Fremont, CA, USA). Sections were incubated with anti-MCT4 polyclonal antibody (anti-SLC16A3, Sigma-Aldrich) and anti-MCT1 polyclonal antibody (anti-SLC16A1, Sigma-Aldrich) at 1/200 and 1/500 dilutions, respectively. Colon mucosa was used as positive control. The omission of the primary antibody was used as a negative control. The TMA sections were incubated with secondary antibody (BioGenex, Kent, UK) for 1 h and then processed using the Polymer-HRP Kit (BioGenex) with development in DAB and contrasted with Mayer's hematoxylin. Scoring was based on intensity and coverage, and ranged between 0 and 300. Two observers (FAM, MB) scored all the cases; the interobserver discrepancy was limited to 3% of cases. These cases were reviewed at the double head microscope, and an agreement was reached.

1.4. In vivo Imaging

When tumor volumes reached 100–150 mm^3 (2–4 weeks following tumor inoculation) mice were transported into a designated SPF laboratory and randomized into 4 groups per tumor model (4–6 mice per cohort) for administration of vehicle, 100 mg/kg AZD3965 by oral gavage (p.o.) for 4h, intraperitoneal injection (i.p.) for 30 min or 50 mg/kg AZD3965 intravenously (i.v.) for 5 min. After treatment, mice were anesthetized with 2% isoflurane/O_2 and scanned on a dedicated small animal PET scanner (G4 Genesis, Sofie Biosciences, Culver city, CA, USA) following a bolus injection of 1.48 MBq of [^{18}F]FDG or 0.925 MBq [^{18}F]-S-FL via a lateral tail vein cannula. After tracer injection, emission scans were acquired in list-mode format [over 0–60 min (dynamic scans) or 40–60 min (static scans) for [^{18}F]FDG; 10–30 min for [^{18}F]-S-FL to give decay-corrected values of radioactivity accumulation in tissues. The collected data were reconstructed with a 3-dimensional maximum likelihood estimation method 3D ML-EM (Sofie Biosciences). Cumulative images of the data were used for visualization of radiotracer uptake and to define tissue volumes of interest (VOIs) using Siemens Inveon Research Workplace software (Siemens Molecular Imaging, Inc. Knoxville, USA). For quantitative comparisons, the count densities of these VOIs were averaged for the time frames corresponding to 40–60 min ([^{18}F]FDG) or 10–30 min ([^{18}F]-S-FL). Tissue radioactivity uptake values were normalized to average whole-body radioactivity. Animals were sacrificed by neck dislocation after imaging. In addition to the imaging studies, biodistribution of [^{18}F]-S-FL was performed 30 min after injection of 0.925 MBq in untreated mice, where tissues were quickly collected following cardiac

puncture and radioactivity content was determined by γ-counting and normalized sample weight. Image analysis was carried out by experienced researchers blinded to treatment and control groups.

1.5. In vivo D-[$^{13}C_6$]glucose and L-[1-^{13}C]lactate Uptake Studies

When tumor volumes reached 100–150 mm^3 (2–4 weeks following tumor inoculation) mice were transported into a designated SPF laboratory and randomized into 2 groups per tumor model (4–6 mice per cohort) for administration of a single injection of AZD3965 at 100 mg/kg or vehicle by p.o. route. At 5 h after drug treatment, mice were slowly injected with 500 mM of a sodium L-[1-^{13}C]lactate (Sigma-Aldrich) solution, pH 7.3, via lateral tail vein; in a similar protocol, 5 h after drug treatment, mice were slowly injected with 500 mM solution of D-[$^{13}C_6$]glucose (Sigma-Aldrich), pH 7.3. Tumor tissue and plasma (via cardiac puncture) samples were obtained at 60 min post-injection of L-[1-^{13}C]lactate and 10 min after D-[$^{13}C_6$]glucose injection and snap-frozen in liquid nitrogen. L-[1-^{13}C]lactate analysis was performed as follows: metabolites were extracted from tumor samples by homogenizing ~60 mg of tumor material in 800µl of cold 80% methanol (−40 °C). Samples were homogenized in screw cap tubes with 0.1 mm glass beads using a Precellys 24 homogenizer (Bertin Technologies, Rockville, MD, USA) operating at 6500 rpm × 20 s × 2 cycles and immediately cooled on dry ice. The resulting extract was centrifuged, and supernatants were transferred to fresh microcentrifuge tubes. The extraction was repeated and supernatants were pooled. Half of the pooled supernatant (800 µL) was taken for GC-MS analysis and dried in a vacuum concentrator. Aqueous metabolites were separated from the intracellular extract using a 2:1:3 chloroform/methanol/water extraction method. The aqueous portion of the extract was separated and dried in a vacuum concentrator before analysis. GC-MS analysis was carried out as previously described. Briefly, 10 µL of 1.5 mg/mL Myristic acid-d27 internal standard was added to each sample and dried in a vacuum concentrator. Samples for stable isotope incorporation analysis were derivatized using a two-step methoximation/silylation procedure. Dried samples were first methoximated with a solution of 20 mg/mL methoxyamine hydrochloride (20 µL) in anhydrous pyridine (Sigma-Aldrich, U.K) at 30 °C for 90 min, then silylated with 80 µL of N-tert- butyldimethylsilyl-N-methyltrifluoroacetamide with 1 % tert-butyldimethylchlorosilane (Thermo Fisher Scientific) at 70 °C for 60 min. After derivatization, 2-fluorobiphenyl in anhydrous pyridine (10 µL, 1 mM) was added to the samples as an injection standard. GC-MS analysis was done on an Agilent 7890 gas chromatograph with a 10 m Duragard integrated with a 30 m DB-5MS capillary column connected to an Agilent 5975 MSD (Agilent Technologies Ltd., Wokingham, UK). Samples were injected with an Agilent 7683 autosampler injector into deactivated splitless liners. GS separation was performed with 1 mL/min constant flow of Helium, oven ramp 60 °C (1 min hold) to 325 °C at 10 °C/min and 10 min hold before cool-down, with a total run time of 37.5 min. GC-MS stable isotope incorporation data were processed with an in-house MatLab package (developed by Dr. Gregory Tredwell) for the quantification of mass isotopomer distributions and correction for natural isotope abundances. D-[$^{13}C_6$]glucose analysis was performed as previously described (Schug et al., 2015). Briefly, tumor material was homogenized. Metabolites from plasma and tumor were extracted with a mixture of methanol, acetonitrile, and water (5:3:2; −20 °C). Following centrifugation (0 °C; 16,000× *g* for 15 min), the supernatant was collected for subsequent analysis. The protein concentration was calculated using a Lowry assay. LC separation was performed using a ZIC-pHILIC column (4.6 × 150 mm, guard column 2.1 × 20 mm; Merck, Watford, UK) eluted with mobile phase comprising formic acid, water, and acetonitrile. Metabolites were detected using Thermo Exactive mass spectrometer (Thermo Fisher Scientific) and analyzed by researchers blinded to treatment and control groups.

2. Supplementary Figures

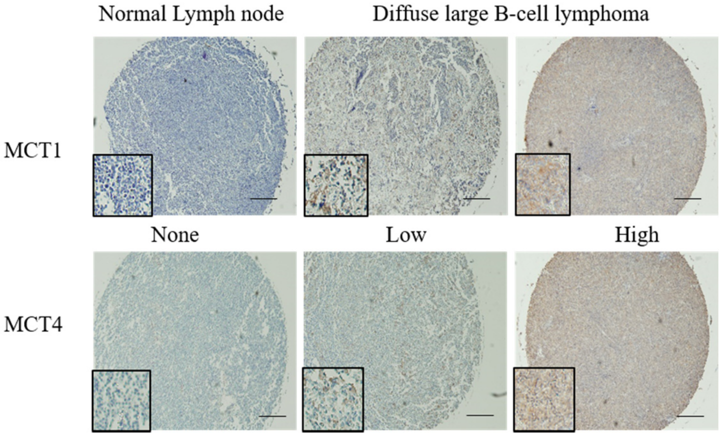

Figure S1. Validation of commercial antibodies for detection of MCT1 and MCT4 in tissue sections. Anti-MCT1 polyclonal antibody (anti-SLC16A1, Sigma-Aldrich) and anti-MCT4 polyclonal antibody (anti-SLC16A3, Sigma-Aldrich) were used at 1/500 and 1/200 dilutions, respectively. Scale bar represents 500 µm.

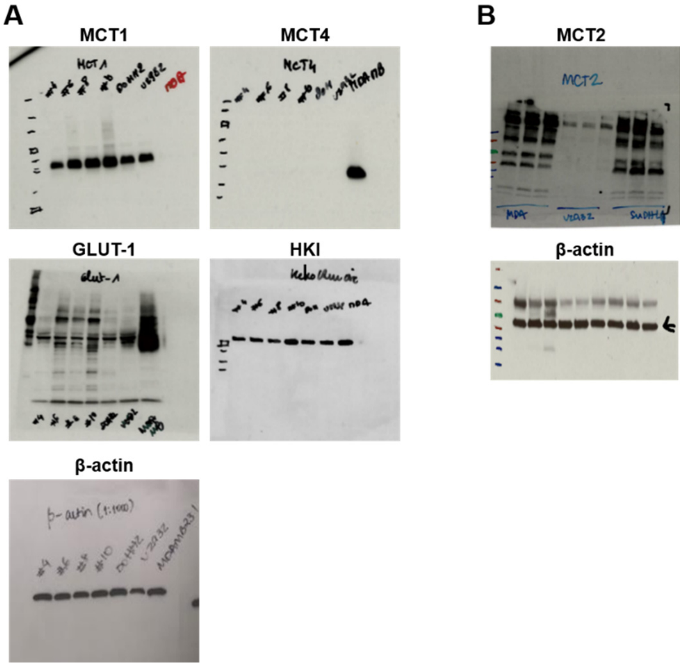

Figure S2. Whole western blots corresponding to cropped bands in (**A**) Figure 1A and (**B**) Figure 1B.

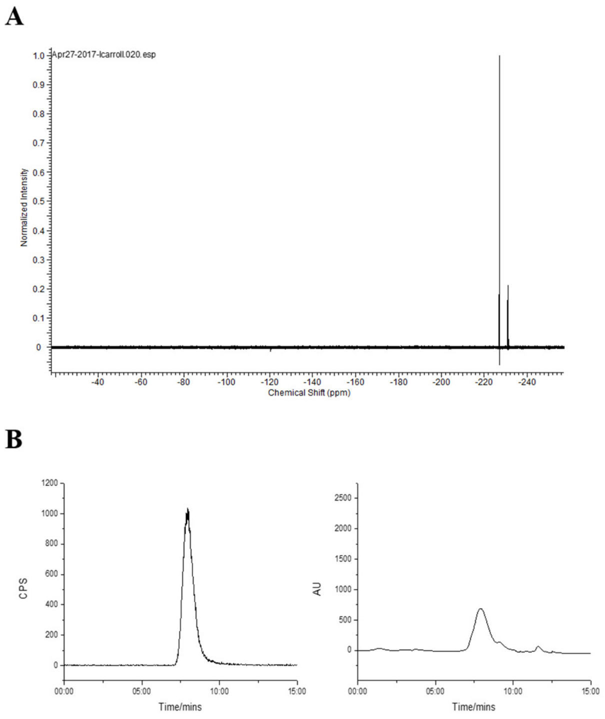

Figure S3. Characterization of [18F]-*S*-FL. (**A**) 19F NMR of reference [19F]-*S*-FL in D20 (376.3 MHz, D20) -229.3 (at *J* = 48.2 Hz), −233.1 (at, *J* = 46.2 Hz). (**B**) Chromatographic analysis of [18F]-*S*-FL showing analytical radio-HPLC of radioactive [18F]-*S*-FL analytical UV-HPLC chromatograms of non-radioactive [19F]-*S*-FL.

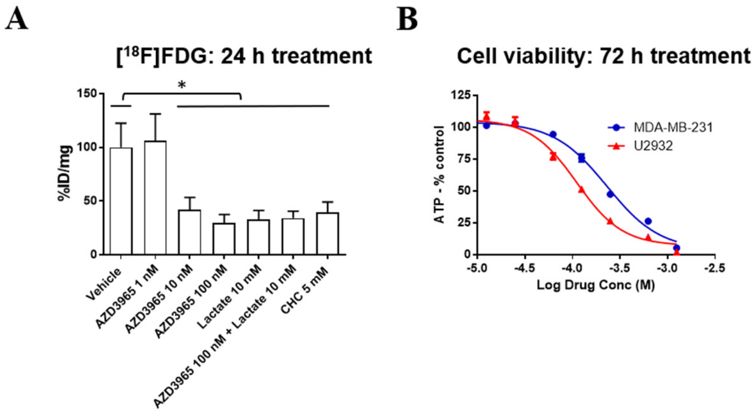

Figure S4. *In vitro* effect of AZD3965 treatment. (**A**) *In vitro* uptake of [18F]FDG (1 h) in U2932 cells following treatment with AZD3965, Sodium L-lactate or α-cyano-4-hydroxycinnamate for 24 h in 2.5 mM glucose RPMI media overnight. (**B**) Dose effect of AZD3965 on cell viability measured using an ATP-glo assay appropriate for both suspension and adherent cells. Cells were incubated continuously with different concentrations of AZD3965 in full RMPI 1640 medium containing 11.1 mM glucose over 72 h (*n* = 4 per concentration). Data were fitted to a sigmoid curve with R2 of ≥ 0.98. IC50 values for U2932 AND MDA-MB-231 were 110.7 and 230.1 nM, respectively. Data are mean ± SEM.

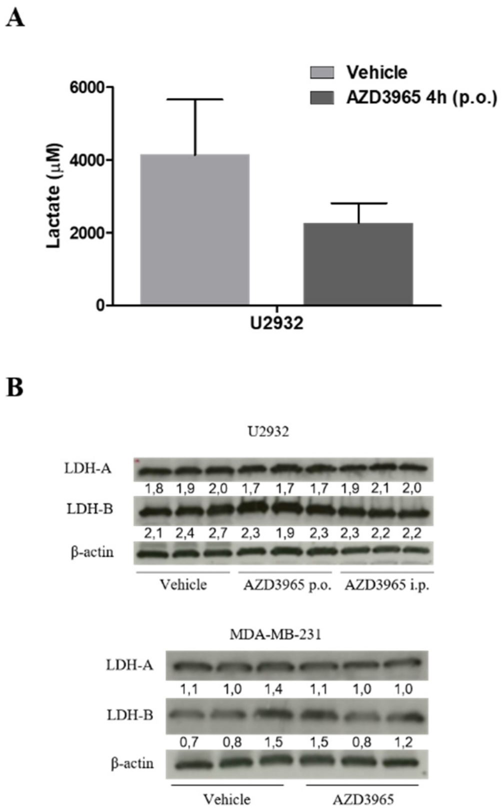

Figure S5. Concentration of lactate in plasma and LDH expression. (**A**) Lactate in plasma of U2932 tumor-bearing mice following treatment with AZD3965 (100 mg/kg, p.o.) for 4 h. Plasma was obtained 10 min after infusion of D-[$^{13}C_6$]glucose (500 nM solution buffered to pH 7.3, delivered at a constant rate of 50 μL/min i.v. via tail vein for 5 min) and analysed by liquid chromatography mass spectrometry. Data are mean ± SEM (*n* = 4). (**B**) Western blot analysis of expression of LDH-A and LDH-B in MDA-MB-231 and U2932 tumors excised from mice treated with AZD3965 (100 mg/kg). Values represent the ratio of protein to β-actin, which was used as loading control; uncropped blots are shown in Figure S10.

A

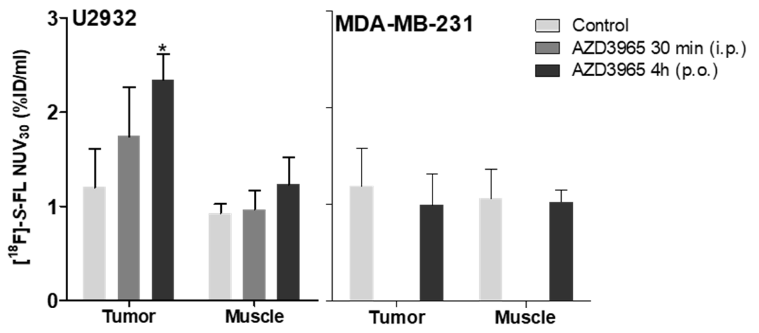

B

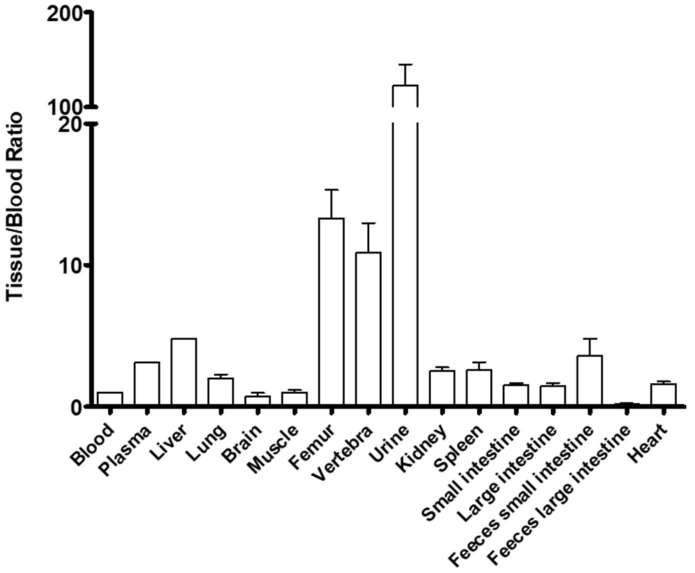

Figure S6. *In vivo* uptake of [^{18}F]-*S*-FL. (**A**) Radioactive normalized uptake value at 30 min post-injection (NUV_{30}) of [^{18}F]-*S*-FL in tumor and muscle after treatment with vehicle, AZD3965 (100 mg/kg) for 30 min (i.p.) and 4 h (p.o.) in U2932 and 4 h (p.o.) only in MDA-MB-231 tumors, expressed in g/mL. (**B**) Biodistribution of [^{18}F]-*S*-FL in non-tumor bearing BALB/c mice at 1h post radiotracer injection. Data are mean ± SEM (n = 4).

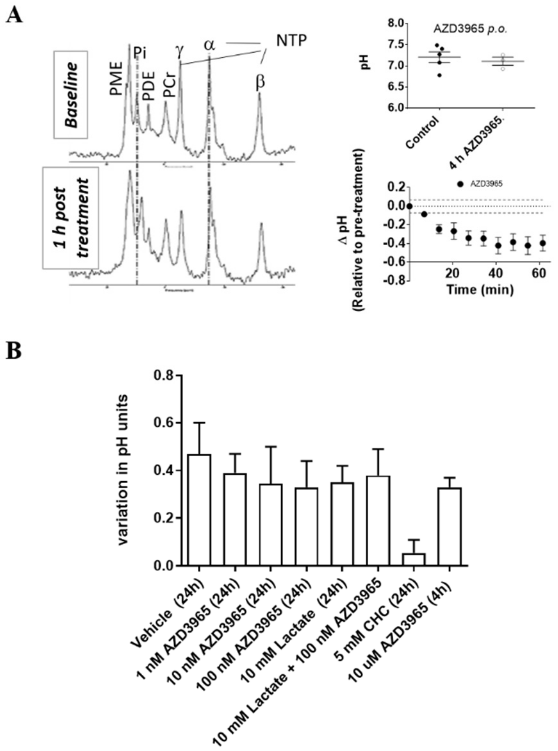

Figure S7. pH variations caused by AZD3965 treatment. (**A**) pH of U2932 tumors in mice treated with AZD3965 (100 mg/kg p.o.) or vehicle assessed by phosphorus magnetic resonance spectroscopy (^{31}P MRS; PME—phosphomonoester; Pi—inorganic phosphate; PDE—phosphodiester; PCr—phosphocreatine; NTP—nucleotide triphosphate; pH was determined from the chemical shift difference between Pi and α-NTP). To examine early changes in pH, a second cohort of mice was treated with AZD3965 (100 mg/kg i.p.). ^{31}P MRS spectra were acquired before drug injection and at multiple times within 1h after drug injection. The broken lines represent change observed in control. (**B**) Effect of AZD3965 and α-cyano-hydroxycinnamate on pH of spent media. U2932 cells were incubated with AZD3965 with or without Sodium L-lactate in 2.5 mM glucose media with pH adjusted to 7.4. The pH of the spent media was measured at 24h. Data are mean decrease in pH ± SEM (n = 4).

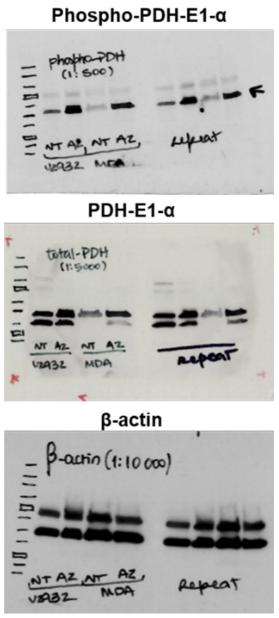

Figure S8. Whole western blots corresponding to cropped bands in Figure 5E.

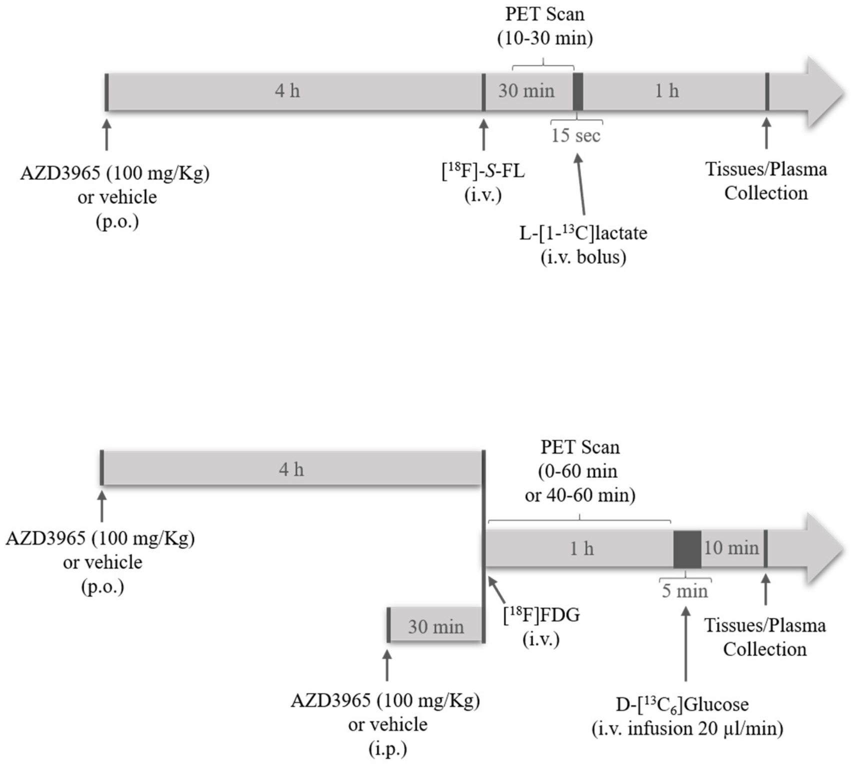

Figure S9. Scheme illustrating timing of *in vivo* experiments involving [^{18}F]FDG, [^{18}F]-*S*-FL, L-[1-^{13}C]lactate and D-[^{13}C$_6$]glucose injections.

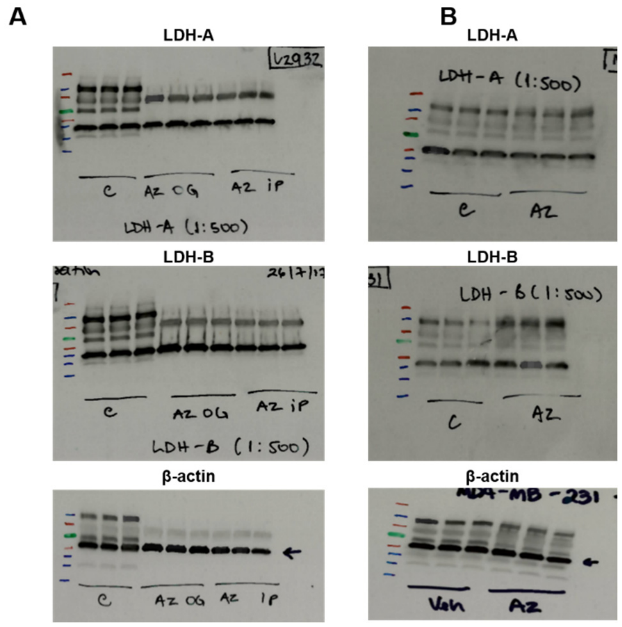

Figure S10. Whole western blots corresponding to cropped bands in Figure S4B. (**A**) Blots corresponding to U2932 and (**B**) MDA-MB-231 tumors.
